# Effective Partnerships Between Local Councils and Health Departments: Lessons From a Disadvantaged Region of Sydney, Australia

**DOI:** 10.34172/ijhpm.9045

**Published:** 2026-03-31

**Authors:** Karla Jaques, Raquiba Jahan Khan, Christopher Browne, Maria Beer, Karen Wardle, Jennie Pry, Susan Gibbeson, Edith Barnes, Tim Hayes, Patrick Harris

**Affiliations:** ^1^Centre for Health Equity Training, Research and Evaluation, University of New South Wales, Sydney, NSW, Australia.; ^2^International Centre for Future Health Systems, Sydney, NSW, Australia.; ^3^Population Health, South Western Sydney Local Health District, Sydney, NSW, Australia.; ^4^Healthy Places, Population Health, South Western Sydney Local Health District, Liverpool, NSW, Australia.; ^5^Collaboration Unit, Population Health, South Western Sydney Local Health District, Liverpool, NSW, Australia.; ^6^Health Promotion, Population Health, South Western Sydney Local Health District, Sydney, NSW, Australia.; ^7^Fairfield City Council, Fairfield, NSW, Australia.; ^8^Wollondilly Shire Council, Picton, NSW, Australia.; ^9^Liverpool City Council, Liverpool, NSW, Australia.; ^10^International Centre for Future Health Systems, University of New South Wales, Sydney, NSW, Australia.

**Keywords:** Social Determinants, Local Government, Health Policy, Intersectoral Partnerships, Collaboration, Australia

## Abstract

**Background:**

Local councils in Australia, established by state governments, are responsible for delivering services, implementing policies, and enforcing regulations that impact community health and well-being. Partnerships between local governments and health agencies can provide a valuable opportunity to advance initiatives to improve health and well-being of communities. This paper explores findings from four case studies of such partnerships in south-west Sydney, Australia.

**Methods:**

Semi-structured qualitative interviews with 25 key stakeholders were conducted as part of a realist evaluation of these partnerships, focusing on factors at functional, organisational, individual, and external levels that influence their success.

**Results:**

The findings provide real-world insights into the enablers and barriers of effective intersectoral partnerships. While the interview data generally align with existing literature and the theory of change developed in earlier research phases, key context-specific differences emerged. The interviews reiterated the need for structural support of the partnerships (in the case studies, through a context specific, locally tailored memorandum of understanding [MoU]) however, structures alone were insufficient, partnerships required "actors" to enable implementation (the partnership officers). Beyond the MoU and partnership officers, wider supports provided by partner organisation through governance, management and workplans were also essential. Stakeholders expressed strong support for these partnerships, citing positive outcomes and the importance of their continuation. However, persistent challenges include sectoral interests and institutional silos that hinder collaboration. The findings underscore the complexity of expecting councils to adopt a health-focused mandate or vice versa.

**Conclusion:**

The study highlights that partnerships, facilitated through MoUs and joint officer appointments, are effective in driving impactful health initiatives. Nevertheless, overcoming organisational silos requires ongoing leadership support and mandates that emphasize the importance of these partnerships. This research emphasizes the critical role of structured collaborations in addressing health determinants and reducing potential inequities within communities.

## Background

**Key Messages**

**Implications for policy makers**

- Formal partnerships between health agencies and local councils improve population health.
- Formal arrangements facilitate effective cross agency working.
- Individuals with core roles supported by organisational structures are necessary.
- The power of siloed sectoral interests is a constant challenge to overcome.

**Implications for the public**

Partnerships between health agencies and local government provide a valuable opportunity to improve population health. This paper applies the international evidence of such partnerships to real world case studies, identifying barriers and enablers of working in partnership to achieve better health and well-being for communities. The case studies demonstrated that these partnerships are transformational for partner organisations, changing the ways of working organisationally whilst also providing collective advantage for partner organisations. However, due to regulatory organisational responsibilities, complete adoption of health into local government agendas and vica versa is not feasible. The study highlights that partnerships, facilitated through formal arrangements (such as memorandum of understanding [MoU]) and individuals with core roles (such as joint officer appointments), are effective in driving impactful health initiatives. Nevertheless, overcoming organisational silos requires ongoing leadership support and mandates that emphasize the importance of these partnerships.

Implementing sustainable partnerships between local government and the health sector to impact on population health is increasingly becoming a priority internationally. However, very limited evidence is available^1-3^ about how partnerships work for effective influence and impact.^3-11^ Studies around the globe support the value of collaborations between health institutions and local government bodies, however, the specific contextual factors as to how these partnerships function successfully (or not) are underdeveloped.^3-5,10^ The factors involved have previously been identified, but how these factors play out in real world local partnerships has yet to be the focus of research. The literature has established that local governments have a role to play in achieving health equity,^11-14^ and partnerships between this sector and health organisations can be an effective (albeit challenging) mechanism to address health equity.^15-17^

 In Australia, the setting for this study, local councils are the layer of government that responds to the needs of environmental health and the well-being of local communities.^1,2,10^ Traditionally local governments provide roads, rates, and garbage services, and they are accountable for the maintenance of built environments including local parks and other community facilities. They are also responsible for the development and implementation of policy and regulations, particularly around built environment, implementing land use planning regulations, and community services that have direct and indirect impacts on the health and well-being of the community.^1^ Australian local health departments tend to focus on providing clinical hospital-based services. Preventative and population-focussed services exist to address the increasing challenges with the wider determinants of health. These services partner with external organisations such as local councils given their role and influence on the determinants of health in local communities. These partnerships are the focus of this article. By linking the international findings to evidence from local partnerships in south west Sydney, Australia, this manuscript provides a practical set of findings about how partnerships between health agencies and local councils work to develop collaborative actions that address health equity.

 The study aimed to evaluate:

1. The ability of the memorandums of understanding (MoUs) to result in indicators for sustainable, equitable health and well-being outcomes;
2. The different roles of health partnership MoUs in building reciprocal capability and collaborative advantage between two different organisations;
3. The functioning and support for co-funded positions between South Western Sydney Local Health District (SWSLHD) and four local councils in south west Sydney to maximise their effectiveness and impact on council and local health district (LHD) business; and
4. Ways to maximise the impact and scalability of the MoUs to other local government areas (LGAs), LHDs and state government authorities.

The research presented here is derived from the second phase of a larger project. Phase 1 of the project focused on establishing a theory of change for health and local government partnerships.^18^ That phase conducted a realist focussed scoping review of the relevant international literature, a document review of four specific partnerships under analysis, and the development of program logics for each partnership. That study categorised four mechanisms that shape the success of partnerships between local government and health agencies (Box 1). This paper focusses on the local experiences of the partnerships in practice.

**Box 1. Mechanisms That Shape Success of Partnerships Between Local Government and Health Organisations^18^**

**Functional aspects of the partnership:** related to the structure and functioning of the partnership itself.

**Organisational factors impacting the partnership:** related to the structure and culture of the organisations in the partnership.

**Individual factors impacting the partnership:** related to agentic factors surrounding the individuals or actors involved in the partnership eg, personalities, skills.

**External factors impacting the partnership:** related to factors outside of the partnership and organisation that have impact on both eg, policy, legislation, local leadership.

###  Case Studies

 The case studies presented in this manuscript are based in the south west of Sydney, Australia. South Western Sydney (SWS) is characterised by a mix of poor health outcomes and socio-environmental risk factors compared to other regions of Sydney. Table 1 provides an overview of relative disadvantage for SWS when compared to the State of New South Wales (NSW) or Greater Sydney.

**Table 1:** Indicators of Relative Disadvantage in Sydney^19-23^

	**SWSLHD**	**NSW**
Born overseas (2016)	43.3%	34.5%
Prevalence of diabetes in adults (2019)	14.5%	11.3%
Current smoking adults (2023)	13.6%	11.7%
Mental health: self-reported high or very high psychological stress (2017)	5%	4.4%
Current vaping >16 years (2023)	8.1%	7.4%
Rental stress (% paying >30% of household income on rent, 2021)	Fairfield 55%; Greenfield Park-Prairiewood 52.3%	Greater Sydney 35.3%
Rate of homelessness per 10 000 (2024)	Fairfield 75.1; Canterbury-Bankstown 72.6	Greater Sydney 49.7
Extreme heat: maximum daily temperatures	8–10.5 degrees hotter than in Sydney CBD	

Abbreviations: SWSLHD, South Western Sydney Local Health District; NSW, New South Wales; CBD, Central Business District.

 SWSLHD, Population Health has had a long-term interest in and partnerships with local councils in the district. The LHD and four (of the seven) councils in its region have developed and signed partnership MoUs, each of which facilitates the employment of a co-funded position to support implementation of joint objectives within each council and the LHD. Three of these partnerships have been established in the last six years, and the other was first established in 1994. The aim and objectives of each of the partnerships differ, as there are tailored MoU agreements for each, however each partnership functions through a “joint position,” a co-funded officer that sits primarily at council but has shared management and/or governance between Council and health. An overview of each of the case study partnerships is provided in Table 2.

**Table 2:** Case Study Characteristics

**Local Councils** **(Population, Land Area, Population Density [Persons Per km**^2^**]** ^24^ ** and LGA SEIFA**^a^**)**	**Partnership Established (y)**	**No. of Staff Interviewed**^b^	**Business Area/ Department Hosting the Joint Position **	**Area of Focus (From MoU Document)**
Campbelltown Population: 184 784 Land area: 311.5 km^2^ Population density: 593.2 LGA SEIFA: 948	2018	5	Social planning and partnerships	1. Embed health and well-being outcomes into Council and NSW Health’s strategic planning, guidelines and policy instruments that apply to neighbourhood and built systems. 2. Guide industry and planners on embedding healthy urban design principles for greenfield developments and urban renewal in Campbelltown LGA. 3. Deliver health related outcomes that are strategically aligned priorities for both SWSLHD and Council to keep people in Campbelltown healthy, well and protected.
Fairfield Population: 209 742 Land area: 101.5 km^2^ Population density: 2066 LGA SEIFA: 814	1994	4	Social planning and community development	1. Contribute to a reduction in chronic disease, promote healthy lifestyles and improve well-being by increasing physical activity and health eating opportunities in the Fairfield LGA. 2. Develop and implement an integrated approach to embedding health and well-being outcomes into Council’s strategic plans in areas such as transport, cultural and social planning. 3. Collaborate to address priority and emerging health, social and built environment issues.
Liverpool Population: 247 672 Land area: 305.8 km^2^ Population density: 809.8 LGA SEIFA: 931	2020	3	Urban design	Key accountabilities and responsibilities will be: (a) addressing the healthy design aspects of: (i) Council’s Community Strategic Plan; and (ii) South West Sydney’s LHD’s: (i) SWSLHD Strategic Plan 2018–2021; and (ii) Population Health Operational Plan for Healthy Communities and Strategic Partnerships. (b) providing urban design expertise and advice that will shape places, spaces, neighbourhoods and streets that drive health and well-being outcomes across the Liverpool LGA; (c) providing Council and SWSLHD with the latest research and approaches to retrofitting and rethinking public places and spaces post the COVID-19 pandemic; (d) leading cross-agency collaboration on a range of Council-led initiatives that will influence sustainability, active transport and physical activity, improved social connectivity, reduced urban temperatures and safe people-oriented places; (e) educating cross-agency peers on how to consider health in urban design and planning processes to achieve better community health outcomes; (f) demonstrating how urban design outcomes support businesses, economic development, prosperity and increased productivity and the relationship to health outcomes; and (g) ensuring urban design outcomes exemplify excellence in design and support multidisciplinary outcomes.
Wollondilly Population: 57 616 Land area: 2556 km^2^ Population density: 22.54 LGA SEIFA: 1041	2018	4	Sustainable growth	1. Develop and implement an integrated approach to embedding health and well-being principles and considerations into Council’s strategic planning, policy development and program delivery. 2. Provide an avenue and foster increased awareness and knowledge of local strategic planning and the strategic planning framework to Population and Health SWSLHD.

Abbreviations: LGA, local government area; MoU, memorandum of understanding; SWSLHD, South Western Sydney Local Health District; NSW, New South Wales; LHD, local health district; ^a^ SEIFA refers to Socio-Economic Indexes for Areas, an Australian Bureau of Statistics measure of relative socio-economic advantage and disadvantage.^25^ ^b^ There were 3 external and 6 health staff interviewed that were not attached to a specific local council.

## Methods

 The study adopts a realist descriptive case study design. In Phase 1 of the project, a critical realist framework was used to identify factors from the international literature that shape partnership activities and outcomes, and to identify potential underlying mechanisms and wider conditions required in effective collaborative working. The current phase maps the results of case study interviews over that evidence-based conceptual framework^18^ and the underlying literature. The focus, in critical realist terminology, is “concretisation and contextualization,”^26^ the purpose of which is to “reapply and test theoretical redescription with real world data”^26^ (p. 44). Our particular focus was on what factors from the literature were activated as being necessary to the working of the partnerships and their impact and outcomes. Critical realist research aims to identify factors that are essential in complex situations, as well as factors that are contingent (ie, they exist as potentially influential but that influence is only triggered under specific conditions).

 A descriptive embedded multiple case study approach following Yin’s Case Study Research Design^27^ provided the comparative design for our realist methodology. We chose a descriptive approach (rather than explanatory – see limitations section in discussion), because the aim was to redescribe the international findings against local experiences. The factors identified in phase 1 became embedded units of analysis to be investigated in that overall multiple case study design. That multiple case study design using the realist underpinnings that focused on mechanisms within particular contexts allowed us to compare and contrast how the findings from that literature played out across the cases, within cases, or within some cases but not others.

###  Study Setting

 A qualitative approach was used to identify the facilitators and barriers of partnerships between local government and health using four case studies in SWS. Our research involved semi-structured interviews with 25 key stakeholders from local government (16), health (6) and externals (3) across four existing partnerships between a local health agency and local government in SWS, Australia.

###  Data Collection 

 Semi-structured interviews were held with key stakeholders related to each of the four partnerships. Interview participants were both internal (directly related to the partnership) or external (no direct involvement but aware of the partnership) stakeholders. The participants were invited via email, the interviews were held online and recorded for transcription using artificial intelligence (AI) technique (Microsoft Teams) and were edited by the research team. The semi-structured interview guides are provided in Supplementary file 1. The document review from phase 1 was reviewed for updates however, no changes were made between phase 1 and phase 2.

###  Data Analysis 

 We took an abductive approach to data analysis that combined deductive (theory testing) and inductive (data emerging from the cases), in line with critical realism. In practical terms, this meant the findings from the international literature provided the a priori framework to test via the cases while simultaneously engaging with the data to explain local experiences.^28^ Data was analysed thematically using NVivo qualitative data analysis software (QSR International Pty Ltd., Version 14, 2023) and was informed by the mechanisms developed in Phase 1 of this project (See Box 1). An NVivo coding framework was developed and tested by KJ and RJK, who had also conducted the interviews. The coding framework was revised and finalised after consulting with PH. Coding was guided by the mechanisms developed in Phase 1 along with thematic themes developed using an inductive method. Data was analysed by KJ and RJK with a sample of 50% double-coding for quality assurance. Any conflicts which arose were discussed to build a consensus. Data was then checked back against the best practice literature established in Phase 1 of this research. This abductive approach deepens the explanation of the findings against existing knowledge and theory, as per critical realist analysis.^26^

## Results

 Results are presented in order of the mechanisms presented in Box 1, along with the enablers and barriers of each. Other additional themes, including partnership outcomes, equity and spread of impact, are also presented. These mechanisms act as pathways towards partnership outcomes, through identifying which factors shape partnership success (or not). Table 3 provides an overview of the findings from the interviews (organised by mechanisms) and whether or not this reflected the best practice literature.^18^

**Table 3:** Interview Results Alignment With Literature

**Mechanism**	**Item (Case Study Findings [Interviews])**	**Literature**	
Functional	Communication and cooperation	✓	Enabler
	Trust	✓	
	Co-funded positions	✓	
	Seeking external grants	✓	
	Reporting to two organisations	✓	
	Multiple levels of governance	✓	
	Flexible context specific	✓	
	Shared measurement of outcomes	✓	
	Advocacy and buy in from multiple levels of management	✓	
	Process as important as outcome	×	
	Spread of impact beyond partnership	×	
	Reporting to two organisations	✓	Barrier
	Multiple levels of governance	✓	
	Differing expectations of workload- power imbalance	✓	
	No funding to complete work	✓	
	Not true commitment, not permanent	✓	
	Long term, hard to quantify health (clinical) outcomes	✓	
	Behaviour change focus	✓	
	Unequal administrative tasks	✓	
	Current funding does not account for inflation	×	
	Co-design is expensive, funding does not allow	×	
	Co-funded position is only one person	×	
Organisational	Aligning with organisational strategic plans	✓	Enabler
	Transformational – new way of working	✓	
	Knowledge and skill sharing	✓	
	Shared language	✓	
	Opportunities to align	✓	
	Change readiness	✓	
	Organisational buy in	×	
	De-siloing, silo buster	✓*	
	Local government organisational context (4-year cycle)	✓*	Barrier
	Lack of institutional buy in	×	
Individual	Skill of shared position	✓	Enabler
	Continuity and champions	✓	
	Networking and shared learning through relationships across different partnerships	×	
	Huge potential but not resources beyond the position (large remit for one person)	×	Barrier
	Trouble with recruitment, short term, large skill set, non-permanent, periods of vacancy	×	
	Reliance on individuals	✓	
	Staffing changes and individuals can unravel partnerships	×	
External	Local government have good relationships with community	✓	Enabler
	State priorities funding opportunities	✓	
	Regional growth issues, lag between growth and infrastructure to support	✓	Barrier
	State policies lack the consideration of health and well-being	✓	

*Note:* ✓ is mentioned in the literature; x is not mentioned; * indicates that the interview finding was the opposite of how it was described in the literature or the interview.

###  Functional Factors 

 Functional aspects related to the way the partnership itself operates were the most frequently discussed mechanism across all the interviews. Across all the partnerships, communication and cooperation were key to their success. Stakeholders expressed the importance of having a clear, collaboratively developed partnership goal/vision as well as formalised governance eg, the MoU, the shared positions and Terms of Reference. This was consistent with the literature, which highlighted the importance of communication,^4,29,30^ governance and shared vision/goals^4,29-32^ as well as flexibility in response to local context.^15,33-35^

 Formalised governance was context-specific, but, crucially and necessarily, needed to be flexible in different applications:

 “*There’s different ways of doing it but I think really the biggest danger for Health would be to think they can have one model and cookie cutter it” *(Council Participant).

 “*So while you can pick the model up, it’s the process that’s important. A committee would need to determine its priorities, its vision, look at its stats. It’s that forming stage that is really important” *(Council Participant).

 Stakeholders acknowledged that the partnerships were built on trust and mutual respect and that this is developed over time. Having that trust in place supported the reality that sometimes necessary frank and honest conversations can be had between partners. The highlighting of the crucial role trust plays in partnerships chimes closely with the literature that also emphasises fostering of trust, transparency and relationship-building.^15,16,4,29,30,32,33,35-37^

 “*I think with our partnership, a lot of it comes down to the trust that’s been established between the organisations and between the people” *(Council Participant).

 Funding of a joint position^30,38^ or allocated human resources^15,31,36,39^ were a common feature of effectively functioning partnerships in the literature. Interviews supported this human resources aspect to the partnerships. A co-funded shared position in each case was explained as critical to the success of each partnership. Importantly, allocating a shared funding and supporting management structures was crucial to support the joint position. Having one person in this role per partnership, situated mainly in council organisations which did not have health as their core business, and occasionally in health organisations that do not have council business as their core business, was something that required navigation by partners. Effort was required to “socialize” the position both internally in each council and to help spread the impact beyond the bounds of the partnership.

 “*The challenge for me is that I don’t feel that I’ve got enough resources in the right places to be able to do everything we could be doing around health and well-being. And I’m really trying to leverage that role as much as I possibly can. But it’s one person with a very specific brief and it is my only health position” *(Council Participant).

 The interviews also suggested that the role of the shared position should be guided toward specific functions and initiatives as there is a risk of the remit being too broad. There was often work to prioritise the focus of the shared position to achieve the most value add and impact.

 “*Well, it’s more just a challenge is it’s a very broad remit of work. So it it’s a lot, we could be involved in so many things. But the problem, the danger there is that if you spread yourself too thin, it’s only one person in this role, they can’t solve every problem that’s out there” *(Council Participant).

 Knowledge, skill-sharing and capacity-building were also highlighted as functional strengths of the partnerships in both the literature^15,30,35-38^ and the case studies. Several stakeholders mentioned the impact that the existence of the partnership itself had on bringing together strengths and skills to achieve targets and goals that would not otherwise be achieved by each partner organisation. Capacity building was a key success of the partnerships.

 “*I think what the health partnership does is give what I call collaborative advantage. Together we achieve more than if we did it separately” *(Council Participant).

 “*The cross influence of organisations is immeasurable. I don’t think it can measure that, so it’s probably more of a story to tell. Because it has been a long journey, but that collaborative advantage is invaluable” *(Council Participant).

 While specificity for the roles was required, the focus of the role required shared goal-setting, rather than rigid emphasis on sector-specific mandates. For instance, the literature highlighted that partnerships which focused on singular health, social outcomes^36^ or resource-intensive behaviour-change programs^4^ were often not successful. This played out interestingly in the case studies, the perception amongst stakeholders being that focusing on embedding health as a strategic issue into the policy level of councils was more beneficial and sustainable for achieving health and well-being outcomes than focussing on distal health outcomes or behaviour change focussed activities. It was acknowledged that whilst behaviour change is important, there is a greater opportunity for impact when policies change to incorporate health and well-being, with the goal of influencing the business of council at an organisational level.

 Many stakeholders highlighted the challenge with local government and health being two very different (government) organisations with different languages and cultures. Some stakeholders mentioned the need to develop a shared language or being “bi-lingual” or “bi-cultural,” learning about how each organisation functions. This was also present in the literature as part of the need for shared understanding of the social, political and organisational contexts of the partners.^4^

 “*Understanding how each other work is something that would have been helpful at the start of the partnership in order to be able to kind of get the most out of it. Instead of spending lots of time explaining to each other” *(Council Participant).

 Differing expectations and perceptions of workloadwere experienced by some of the partnerships. In some cases, this led to a power imbalance and hierarchical relationships driven by sectoral rather than cross-sectoral demands, which was detrimental to the partnership. This was consistent with the literature, with workload imbalances and poor management of partnership administration leading to challenges in partnerships.^15,31^ It was highlighted that administrative tasks including the constant need to renew the MoU can get in the way of time dedicated to delivering outcomes and, at times, undermine these intended outcomes. Reporting to two organisations raised some issues, being time-consuming, confusing and ultimately taking time away from producing work.

 “*Their [shared positions’] time is valuable. We just need to think about how we get that joint position working and what they’re working on to get the biggest bang for their buck” *(Health Participant).

 “*Unnecessary layers of hierarchy… Some of the partnerships are over-managed cause there’s too many tiers... I don’t think this triangular kind of hierarchical reporting system is necessary” *(Health Participant).

 Multiple levels of governance of the broader partnership was also a challenge for meeting objectives. While supportive of the co-funding going towards the shared position, it also meant that there was not sustainable funding in place to deliver the work, with partners often having to seek external funds to sustain their activities. There was a willingness from partners to secure external funds through grant processes and this was viewed as an enabling factor of the partnerships and within the literature.^15,40^

 “*Because without the joint funding, it wouldn’t have the profile it has quite simply. Let’s just call it that for what it is” *(Council Participant).

 The literature identifies that a lack of long-term sustainable funding challenges these partnerships.^4,15,29,31,38,41^ This challenge became apparent in the case studies. The current funding model has not changed in a number of years, so does not account for inflation or on-costs and was seen as a relatively decreasing amount which affected the ability to recruit to the position. It was argued that the renewal of the MoU every three years was in conflict with the concept of embedding health into policies and, ultimately, into communities. That impact, it was highlighted, was a long-term endeavour. True commitment was viewed as making the positions permanent with a particular commitment from Health to deliver health outcomes through their local councils.

###  Organisational Factors 

 Organisational aspects relating to the structure and culture of the partner organisations and the way these interact were also common in the case studies. Alignment of organisational objectives^4^ and areas of overlap^17^ were also highlighted within the literature as enablers. Stakeholders in the cases understood the necessity of considering the sets of objectives of both partners. There was work involved in connecting those objectives: looking for opportunities to align with each organisation’s strategic plans and implementing shared activities to achieve these goals were at the heart of that work.

 Strong formal and informal leadership providing advocacy for partnerships^38^ was highlighted in the literature as crucial. Organisational buy-in to the partnerships was mentioned as a key element of success across the case study partnerships. In some cases, it was felt that buy-in from higher levels was beneficial as it improved visibility and value for the partnership, however this was not always essential. Political will or buy-in were identified as critical to some partnerships in the literature for both facilitating partnerships and securing funding.^30,42^

 “*The MoU really, and the governance arrangement it’s great to have at a level where it has visibility for the most senior people in Council, Chief Executive, the Mayor and people on the Executive for Council. They may not be involved in the ‘doing,’ but having that kind of visibility I think is great” *(Health Participant).

 A number of participants highlighted existing relationships and networks within local communities as supporting the partnerships. However, it was also raised that without adequate resourcing, it was difficult to work “with” community to delivery outcomes rather than doing things “to” the community. True collaboration and co-design with communities is costly, requiring time and resources which is something the current model does not always allow for.

 “*I think there’s limited room for trial and error. As a person on the other side, you have to feel like you’re being involved or your opinions matter and are incorporated. So, all of that takes a lot of effort and listening and I think that is not facilitated to the extent that it could be given the time and resources that we have. You have to make do with what you’ve got, and that doesn’t always like allow programmes to reach the potential that they could” *(Council Participant).

 Change readiness was a key organisational enabler identified in the literature.^15,32,37,38,43^ Participants described how the partnerships were transformational for their organisations, given that they were a completely new way of working. This was particularly the case with councils, where, in the absence of the partnership, participants explained, health would not be on their agendas. Participants indicated that the partnership approach was moving forward building on each partner’s knowledge and skills. This positive cross-agency readiness for change reflected best practice in collaboration and reciprocity.^15,32,37,38,43^

 “*One of the uniqueness’s of partnerships is it’s transformational. Its actually mobilised the both Health and Council in putting health on the agenda, which has long-term benefits and without the partnership, neither organisation would be where we are” *(Council Participant).

 However, less positively, the political realities of working in local government challenged the partnership. The politics of councils was raised as a challenge in each case study, as Sydney councils undergo elections every four years, which tends to shift focus away from strategic engagement to more local concerns over the core role of councils. This was reflected in the literature, with bipartisan politics and sector reorganisation a consistent challenge to intersectoral partnerships.^29,30,36,39,40^ Health is similarly subject to state level changes of politics however, this was not raised by the participants.

 “*In local government, we obviously have elections [every 4 years]. So, we can’t commit a future Council to funding” *(Council Participant).

 Strategically, councils are organisationally bound to the process of developing Community Strategic Plans based on community priorities. Participants mentioned the opportunity to align partnership activities to such documents and associated processes to enhance and embed the partnership and its work further.

 “*And equally, I think it’s really important for the officers within Health who are working with Council to understand the importance of the Community Strategic Plan, which is actually not Council’s vision for the future of the LGA. It’s the community’s vision and it dictates everything that we do” *(Council Participant).

 Political and structural differences between the partner organisations were also explained as requiring active navigation and bridging. Organisationally the two partners are very different, and a shared understanding that develops over time was important for reciprocal understanding of how each organisation functions. One council gave a particular example of a point of difference between the partnership and council, in this case advocacy for a hospital. That funding was not something that the partnership could support, but was a political aspiration that the shared position in each case study, as well as the steering committees involved, regularly had to navigate.

 Three of the four partnerships focussed on healthy urban planning. However, the scope and approach of “planning” within those councils was highlighted as both a potential enabler and barrier to the partnerships. One participant described how there was a difference in planners’ views of “planning” and the demand of the council on the role of planning and planners. Some had an expansive, diverse view of planning while others had a more conservative view, seeing it as more constrained to land use.

 “*What I’m saying is, [specific council’s] approach to planning...has been a fairly conservative view of land use planning... Other planners aren’t onboard with that view (being expansive and progressive)” *(Council Participant).

 It was acknowledged by a number of stakeholders that there was huge potential for influence considering health and well-being within local government. However a number of participants mentioned a lack of wider institutionalised commitment, beyond the partnership, to health and well-being, a point that was different within the literature, with strategies such as Health in All Policies being an enabler of health equity as an organisational goal.^16^ The siloed nature of specific sectors is identified in the literature as the fundamental challenge to health and well-being focussed partnerships. The case studies suggested that the goal can never be to override those siloed sectoral demands (which are historically and institutionally embedded in policy systems), but instead, the whole aim of a partnership is to bridge those siloes to arrive at collaborative solutions to long-standing problems like health inequities. However, the nature of that partnership work, within a siloed policy context, was a constant tension that required navigation.

###  Individual Factors

 Individual factors zeroed in on the people involved in these partnerships and how they contributed to their effectiveness. Some participants felt that, while there are huge opportunities for health and well-being within local government, these opportunities are likely to be missed if the partnerships are not resourced enough beyond the shared positions. They argued that there were too many possible areas of work to be handled by a single staff member per council, irrespective of how highly skilled this individual was.

 “*We could be involved in so many things. But the danger there is that if you spread yourself too thin it’s only one person in this role, they can’t solve every problem that’s out there”* (Council Participant).

 Continuity of individuals within partner organisations was highly valued by participants and was an enabler of partnerships in the literature.^4^ The ability of the shared positions to upskill others, particularly within the council organisations, was highly valued to spread and extend the impact of the role. There were examples of the positive impact of having long-standing members, as well as recognition of how specific members of the partnerships have driven their success. Other participants reflected on the partnerships’ ability to change the way people think, conceptualise and approach their work.

 “*It’s just opened up a whole new world for me, I’ve learnt a lot more about health that I never knew before. I didn’t know how it operated, who we need to speak to, where we needed to go, etcetera. So, it’s all those little things that are of a huge benefit as well” *(Council Participant).

 The fact that there are four of these shared positions in the geographical area meant that the shared positions were able to collaborate with their co-officers and share learnings on an informal basis.

 While the existence of champions was an enabler of success for some partnerships (and was also present in the literature^4^), an over-reliance on individuals rather than structures built within an organisation was identified as a risk to the partnerships. That is, if an individual in a shared position role has a particular background/passion/skill, that has the potential to be lost if they were to move on.

 Across several partnerships, there were difficulties experienced with recruiting to the shared positions. This was attributed to multiple factors including the specific skill set that is required (covering two sectors; health and planning), to influence stakeholders and actively drive change, the high demand for planners, and non-permanent contracts due to constant re-negotiation and pushes to provide a business case for the continued roles. Staff turn-over was relatively high in two of the councils compared with the other two. That turnover was explained as losing capacity and knowledge when staff move on as well as the need to retrain when new people come on board. A specific issue raised was that periods when the positions lay vacant were not accounted for in the MoU’s timeframe or financial contribution. Risks accompanying staff turnover were suggested by some participants as evidence of the fragile nature of the partnerships.

 “*If [shared position officer] left tomorrow, we’d probably struggle to get someone of their capability again. And (we’d) be back to square one of when I started, when it was vacant for six months” *(Council Participant).

 “*So in terms of trying to recruit people and keep people in these positions, it’s really hard to get someone of quality to do the sort of work that we want with only $100,000 on the table. Particularly when planners and designers are in really short supply, it’s a really competitive arena” *(Health Participant).

 “*[We are] Trying very hard to find the right people to fill up this role. Because it’s a unique role in itself, where we want somebody who is quite design-focused and also has some experience or inclination towards healthy placemaking. And that’s where, because one remit of it is quite new and it’s an evolving sort of discipline in itself” *(Council Participant).

 “*They’re [partnerships] also super fragile. When the staff changes, they will often change as well… and it’s not just about the person, it’s about the cultural change they impart on their organisation” *(External Participant).

###  External Factors

 External, contingent, factors that lie beyond the control of partner organisations were identified that nevertheless had an impact on the partnerships.

 Some participants expressed that ideologically, health and well-being is well accepted across various levels of government. That support for health as an idea was viewed as an enabler that strengthens the need for the (partnerships) to occur.

 Similarly at a macro level, there was broad cross-government acceptance that south west Sydney has some of the fastest population growth in NSW (which in fact is 1.5 x the NSW growth^44^) and that this is not in line with growth in services and infrastructure. The planning and implementation of these services and infrastructure were generally beyond the control of local government, making it challenging for councils to meet the needs of the local community.

 “*One of the key challenges that we have at the moment is the quality and amount of infrastructure that’s available to support the population, particularly as we’re growing quite substantially… So having that lag between population growth and the services and the infrastructure to support that population, particularly when we’ve got quite disadvantaged populations, it is an issue” *(Council Participant).

 The literature highlighted the challenge of conflicting sectoral agendas at higher levels of government.^4,32^ Some of the partnerships, particularly those focused on planning outcomes, expressed that a lot of what is trying to be achieved/addressed is controlled by the State government, beyond the scope of what local government can influence or change. It was also highlighted that, currently, State government policies within planning simply do not consider health and well-being, an ongoing challenge to the work of the partnerships. However, there were examples where State government priorities aligned with health and well-being and were therefore an opportunity for the partnership to apply for grants. The literature also highlighted the opportunity for effectiveness when national policy or legislation aligned with partnership goals.^4,32-34,36^

###  Partnership Outcomes 

 As is established in the literature, evaluation that includes accountability and measures of success is important in the functioning and eventual success or failure of a partnership. Stakeholders highlighted the importance of incorporating measurement plans for each project in the MoU, including specific outcome indicators from a health and well-being point of view. All of the partnerships had mechanisms for monitoring and evaluating the work of the partnership: while this was usually guided by the high-level MoU, the more detailed description on objectives and outcomes of the partnership usually took the form of work plans, operational plans or program logics. This was reflective of the literature, with agreed, shared measurement systems of success important for the functioning of successful partnerships^30,31^ as well as the application of theory of change (program logics) as an evaluation tool.^38^

 The literature highlighted the challenges with evaluating partnerships, including the use of longer-term goals that may not have clear, easily attributable outcomes.^16,34^ Council participants stated that they are eager to continue working with health, and they appreciated embedding health in built environment, however expressed concerns regarding the time required to measure the set outcomes and engaging with factors such as organisational and partnership key performance indicators. Some participants also felt that measuring outcomes in terms of health and well-being was a challenge.

 “*The short answer is yes, but it is hard to quantify. I think we are playing the long game, we are chipping away, being an advocate on incremental behaviour change. These are not going to happen immediately, or overnight, time and consistency is needed to deliver change that eventually we see in terms of community outcomes” *(Council Participant).

 Investment of time was a major issue raised by a number of stakeholders that is, the time it takes to deliver health and well-being outcomes in the community. Many of the “outcomes” of the initiatives being implemented had very long-term health and well-being outcomes; this was particularly the case in the health in planning focused partnerships. The literature likewise highlighted that partnerships with a very long-term focus and no clear or achievable outcomes were not able to demonstrate tangible outcomes.^16,34^

 Where the co-funded position sits (department) within council and its primary focus was also highlighted as key to how and what outcomes could be measured.

 “*It’s a bit of a challenge and again, it’s limited capacity. Because we don’t do the recreation and open space planning. It does make it a little bit difficult for us to sort of have any sort of influence in that space” *(Council Participant).

 There was also some discussion about differences in language/articulation of what would be considered an outcome for each of the partner organisations. Some participants highlighted that it can be a balancing act between pursuing big picture outcomes and being focused enough to provide outcomes in the shorter term. This was not always seen as a challenge but an issue that needed to be navigated. The literature also highlighted the importance of agreement on what should and should not be considered evidence, whether that be service delivery measures, network analysis, integration of health into policies, or health outcomes.

 “*We are always kind of walking that fine line between what we see as an outcome and what a Council sees as an outcome and Councils see process as outcomes” *(Health Participant).

 Other participants highlighted the constraints of ‘clinical’ health measures of success, with that focus setting the partnerships up for failure as such measures take too long to become apparent (beyond any MoU agreement) and may be impossible to directly correlate to the partnership’s work. Studies in the literature review also highlighted this, expressing difficulty in isolating causation of outcomes to the partnerships themselves (as opposed to being an enabler for the delivery of outcomes in a broader context).

 Participants also highlighted that process is just as important as outcomes. For example, the integration of health into planning and policy documents is just as important an outcome as running a successful healthy eating event or increasing tree canopy in an area. One participant expressed that in integrating health into council plans, it would improve the submission process in that Health would no longer have to comment as much on submissions, as Health’s priorities would be addressed much earlier in the planning cycle.

###  Equity

 When stakeholders were asked about whether Health Equity was considered at all in terms of outcomes of the partnerships, most participants mentioned that while it is typically considered it is not explicitly incorporated into the partnerships or MoU agreements. This was similar in the literature, with an example from the Netherlands noting poor awareness or lack of prioritisation of health equity amongst policy-makers.^17^ Some participants indicated that local councils tended to consider equity in the way that they prioritise and deliver services more generally, but again it was not explicit. There were some examples of partnership activities where equity was implicit such as the ‘Healthy Streets’ approach which weights assessment of streets according to disadvantage or poorly prioritised areas.

###  Spread of Impact

 A large number of participants highlighted the innovative model that has been developed and implemented in SWS, in particular the co-funded officer model. This model has been scaled up to different councils in the area and is being explored in an additional council (in partnership with another LHD) as well as at the state level (eg, Transport for NSW). Within organisations, there has been “spread” of impact beyond the bounds of the partnership, ranging from awareness among other staff and teams to changes in career paths.

 “*It’s almost been a bit of an awareness piece for other staff within the Council and I hope the Community too. I have lined up [staff member external to the partnership] to get some mentoring and exposure to some of the work that the position does, so we are now doing shared learnings within [the organisation]. It’s also encouraging others to maybe go down different career pathways too. So that’s been a benefit” *(Council Participant).

 “*We’re starting to see a commitment of Council through budget allocation for healthy urban planning and design work. When Council then goes over and above that (co-funding the position) and commits to a recommendation. I think that’s an outcome in my book” *(Health Participant).

## Discussion

 This study describes the experiences and perspectives of four case study examples of partnerships between local government and a health agency in south west Sydney, Australia. Using a theory of change approach, we explicitly linked evidence from those four cases against the international literature about health and local government partnerships across functional, organisational, individual, and external factors. We have documented the factors that were supported by the local evidence, those that were not, and those that either contradicted or were the inverse of the international literature.

 For practice, a core set of findings emerged about local level partnerships and what has become known as healthy public policy or Health in All Policies. That literature stresses the need for structural level support that provides institutional mandates for action.^45-47^ We showed that an overarching MoU between the health agency and each local government in part provided that structural imprimatur. Importantly, each MoU was tailored to the specific interests of that council in progressing actions and work to positively impact health and well-being. Structures alone are insufficient, however, and require individual actors to develop and implement action. The implementation of each of those agreements was supported by investment in an officer in each council, working under a specific team in each council and connected back into a team in the local health organisation. Crucially, however, alone the MoUs and officers were not the main mechanisms for effectiveness. That came about through wider supports provided by each organisation involved in the partnership. These supports included governance and management, against a clear set of workplans, goals and objectives. In terms of addressing health equity through these partnerships, our case studies highlighted the need for its explicit inclusion within governance structures: otherwise it is simply not considered, which aligns with literature highlighting the challenges of the consideration of equity in public health interventions.^48^

 Crucially, with an institutional lens, it became clear that wider organisational support was needed because ultimately the organisations involved are oriented towards specific sectoral, regulated, roles and responsibilities.^49^ For instance, recent Australian research has shown the challenges of local governments recognising the determinants of health.^11^ That pressure to work for specific sectoral ends constantly risked the partnerships which were, ultimately, designed to cross those sectoral boundaries. Without continued organisational level leadership and support, partnerships are at risk of being crushed by the very institutional forces they aim to overcome. Shared capacity-building activities were also key to broader organisational impacts for example, applied training incorporating both health and local government was an example of a strategy to spread the impact beyond the boundaries of the partnerships or the shared officers. Although not possible in the current context of the 4 case studies, stakeholders raised concerns over the temporary nature of the funding of the shared positions and the need to re-negotiate the MoU every 3 years, both of these being in conflict with the concept of embedding health into policies. For practice the recommendation was shared positions should be made permanent to ensure sustainability of embedding health into policy as well as the ability to assess health impacts of the partnership in the longer term.

 For research, we have shown the value in taking a realist lens to unpack the practical balancing of factors that make partnerships function in the face of institutional forces that are difficult, if not impossible, to shift. Isolating the many factors at play in a cross-organisational partnership, and then stratifying these against wider structural forces outside the control of those organisations, provides a valuable lens for the reality of the partnership working. The notion of creating new “spaces” (which in the case studies were the partnerships) which bring together people, ideas and influence in the face of institutional inertia is a feature of the literature on power and policy and has been shown to be necessary to influence policy systems to be more equitable.^50,51^ Institutional silos are always going to exist, the partnerships create what is known in the power and policy literature as “spaces” to progress novel work that crosses sectors. The dynamics of how those spaces work has been the focus of this paper (demonstrated in Table 3). Our findings and analysis are reflective of broader work on governance and policy^52^ and specifically, health in all policies.^45,53^ We contend that the nature of our inquiry is sufficient to progress future research into how local partnerships across a range of settings work to improve population health outcomes. Realist evaluation is aligning itself with systems-focussed research^54^ given the complexity of the forces at play in complex interventions like partnerships. Similarly, here we demonstrated how systemic complexity can be unpacked by linking the international literature against local evidence about what works and why in the development and implementation of partnerships between the health and local government sectors.

###  Limitations 

 The study is not without limitations. We took a descriptive lens to the data rather than a critical theory approach. That aligns in part, but not fully, with a critical realist methodology in that we were able to connect local experiences to deeper explanations from the international literature in a mix of inductive and deductive analysis (termed abduction). However, we did not situate that analysis in a deep explanation of why various findings came about. While this is a limitation for critical realists, our aim was more empirical and practice-focussed than a critical analysis of that practice. Further, our sample is small and analysis was qualitative, which restricts generalisability. However, we did demonstrate how the range of factors from a number of global studies played out at a local level, which provides important internal and external validity for our analysis, in line with best practice case study methodology.^27^ This study took a descriptive approach because our aim was to redescribe the international findings against local experiences. The formative phase of this study (scoping review) used an exploratory approach, describing the core factors that influence successful partnerships between local government and health agencies. This study built upon this exploratory approach (the conceptual framework), situating the core factors against real world examples. There is potential for bias in our small sample of self-reported perceptions of the various partnerships however the subjectivity is the nature of qualitative research.

## Conclusions

 This qualitative research describes multiple real-world examples of barriers and enablers when local government and health agencies work in partnership to achieve better health and well-being for communities. This paper established the functional, organisational, individual and external level aspects that drive the effectiveness of these partnerships between a health agency and local government. It has also explored the key considerations for measuring outcomes in the local application of these partnerships. Generally, the information from the stakeholder interviews aligns with the literature which informed the theory of change in Phase 1, with the exception of some key context-specific differences. The stakeholder interviews found overwhelmingly positive responses regarding the partnerships and support for them to continue. The application of the model in each of the LGAs is innovative and has been transformational for organisations, changing the ways of working organisationally. The partnerships provided collective advantage for partner organisations by working together. Partnerships can come about through an interest within councils in health, and within Health departments an interest in councils, and the partnership maintains that interest. In other instances, the partnerships led to health being on the agenda of local governments and local government being on the agenda of health. However, due to regulatory organisational responsibilities, complete adoption of health into local government agendas is not feasible. It is also not feasible to expect that one position (shared officer) would result in this complete adoption, again due to dual organisational responsibilities. However, building support for the integration of health around this shared position, and “socialising” this role, rather than relying solely on its existence, results in organisational change.

## Acknowledgements

 We would like to thank the interview participants for their valuable contributions to this study.

## Disclosure of artificial intelligence (AI) use

 Microsoft Copilot, an AI tool, was used during the revision process to assist with formatting of newly inserted references.

## Ethical issues

 This project was considered a quality improvement project by the SWSLHD Research and Ethics Office so was not required to undergo ethical approval.

## Conflicts of interest

 Authors declare that they have no conflicts of interest.

## Supplementary files


Supplementary file 1. Semi Structured Interview Guide.
