# Supplementary material for: Effective Partnerships Between Local Councils and Health Departments: Lessons From a Disadvantaged Region of Sydney, Australia
Source: Int J Health Policy Manag. 2026 Mar 31;15:9045. doi: 10.34172/ijhpm.9045 (PMC13338731; doi:10.34172/ijhpm.9045)
Supplement: Supplementary file 1 — Semi Structured Interview Guide. [file ijhpm-15-9045-s001.pdf]

Article title: Effective Partnerships Between Local Councils and Health Departments: Lessons From a Disadvantaged Region of Sydney, Australia

Journal name: International Journal of Health Policy and Management (IJHPM)

Authors' information: Karla Jaques[1,2,3]*, Raquiba Jahan Khan[3], Christopher Browne[4], Maria Beer[5], Karen Wardle[6], Jennie Pry[4], Susan Gibbeson[7], Edith Barnes[8], Tim Hayes[9], Patrick Harris[10]

[1]Centre for Health Equity Training, Research and Evaluation, University of New South Wales, Sydney, NSW, Australia.

[2]International Centre for Future Health Systems, Sydney, NSW, Australia.

[3]Population Health, South Western Sydney Local Health District, Sydney, NSW, Australia.

[4]Healthy Places, Population Health, South Western Sydney Local Health District, Liverpool, NSW, Australia.

[5]Collaboration Unit, Population Health, South Western Sydney Local Health District, Liverpool, NSW, Australia.

[6]Health Promotion, Population Health, South Western Sydney Local Health District, Sydney, NSW, Australia.

[7]Fairfield City Council, Fairfield, NSW, Australia.

[8]Wollondilly Shire Council, Picton, NSW, Australia.

[9]Liverpool City Council, Liverpool, NSW, Australia.

[10]International Centre for Future Health Systems, University of New South Wales, Sydney, NSW, Australia.

***Correspondence to:** Karla Jaques; Email: k.jaques@unsw.edu.au

Citation: Jaques K, Khan RJ, Browne C, et al. Effective partnerships between local councils and health departments: lessons from a disadvantaged region of Sydney, Australia. Int J Health Policy Manag. 2025;14:9045. doi:10.34172/ijhpm.9045

Supplementary file 1. Semi Structured Interview Guide

Internal Stakeholders	External Stakeholders
1. Background (discipline, years of working, role, relation to the partnerships [note which one])<br>2. What do you think the Partnership[s] are trying to achieve in terms of outcomes for health and wellbeing?<br>3. Have they been successful in this?<br>a. If so, how (give examples)<br>b. If not, why not?<br>4. What contributes (or limits) to the success in terms of health and wellbeing outcomes?<br>5. Are there any set indicators for health & wellbeing outcomes?<br>a. If yes, what are these?<br>6. Can you please talk about the issues of sustainability, do you think the outcomes are sustainable?<br>7. Is equity/health equity considered at all in terms of outcomes?<br>a. Do you think there are equitable outcomes?<br>8. Do you think the partnership[s] contributes to develop, and facilitates a better reciprocal capability and collaboration for each partner organisation?<br>a. If yes, how? (give examples)<br>b. If no, why?<br>9. Are these capabilities and collaborations shared equally between organisations?<br>10. In terms of the partnerships functioning, what do you think is working	1. Background (discipline, years of working, role, relation to partnership[s])<br>2. What has been your involvement in the partnerships between councils and the health sector in SWS?<br>3. What do you think the partnership[s] are trying to achieve in terms of outcomes for health and wellbeing in South Western Sydney?<br>4. Do you think the partnership[s] have been effective?<br>a. If yes, how?<br>b. If no, why not?<br>5. Do you think this partnership[s] has the potential to work elsewhere or be scaled up?<br>a. Other local government/health district setting, and<br>b. State and federal level.<br>6. Is there anyone who you would suggest we talk to about this?<br>7. Are you aware of any similar partnership arrangements between local councils and other agencies that co-fund joint positions?

well/contributing to success or not working well?<br>a. Please talk about what is not working well (limiting success) and why/how.<br>b. If it is working well, why/how?<br>11. In your opinion, how does the partnership impact the Council business and Health District business (e.g., policy, practices and programs)?<br>12. Do you think this partnership model has the potential to work elsewhere?<br>a. For example, in other local government/health district settings, and State.<br>13. Are you aware if other LHDs are working in partnership with their local councils to improve population health outcomes?<br>a. If so, please describe.	
